# The outpost against cancer: universal cancer only markers

**DOI:** 10.20892/j.issn.2095-3941.2023.0313

**Published:** 2023-11-28

**Authors:** Chengchen Qian, Xiaolong Zou, Wei Li, Yinshan Li, Wenqiang Yu

**Affiliations:** 1 Shanghai Epiprobe Biotechnology Co., Ltd, Shanghai 200233, China; 2 Department of General Surgery, the First Affiliated Hospital of Harbin Medical University, Harbin 150001, China; 3 Shandong Epiprobe Medical Laboratory Co., Ltd, Heze 274108, China; 4 People’s Hospital of Ningxia Hui Autonomous Region, Ningxia Medical University, Yinchuan 750002, China; 5 Shanghai Public Health Clinical Center & Department of General Surgery, Huashan Hospital & Cancer Metastasis Institute & Laboratory of RNA Epigenetics, Institutes of Biomedical Sciences, Shanghai Medical College, Fudan University, Shanghai 200032, China

**Keywords:** Cancer detection, cancer screening, DNA methylation, cancer epigenetics, cancer biomarkers

## Abstract

Cancer is the leading cause of death worldwide. Early detection of cancer can lower the mortality of all types of cancer; however, effective early-detection biomarkers are lacking for most types of cancers. DNA methylation has always been a major target of interest because DNA methylation usually occurs before other detectable genetic changes. While investigating the common features of cancer using a novel guide positioning sequencing for DNA methylation, a series of universal cancer only markers (UCOMs) have emerged as strong candidates for effective and accurate early detection of cancer. While the clinical value of current cancer biomarkers is diminished by low sensitivity and/or low specificity, the unique characteristics of UCOMs ensure clinically meaningful results. Validation of the clinical potential of UCOMs in lung, cervical, endometrial, and urothelial cancers further supports the application of UCOMs in multiple cancer types and various clinical scenarios. In fact, the applications of UCOMs are currently under active investigation with further evaluation in the early detection of cancer, auxiliary diagnosis, treatment efficacy, and recurrence monitoring. The molecular mechanisms by which UCOMs detect cancers are the next important topics to be investigated. The application of UCOMs in real-world scenarios also requires implementation and refinement.

## Why we urgently need new biomarkers?

After combatting cancer for over a century, cancer is still the most lethal biological threat to mankind. Cancer remains a global health concern with 19.3 million new cases and nearly 10 million deaths estimated in 2020^1^. In 2020 an estimated 4.6 million new cases of cancer were diagnosed in China, accounting for 23.7% of new cancer cases globally according to GLOBOCAN^1^. Furthermore, approximately 3 million deaths were attributed to cancer in China in 2020, which were 30% of global cancer-related deaths^1^. These statistics indicated that China ranks first in the incidence and mortality rate of cancer. Moreover, the 5-year cancer survival rate is 40.5%, which is 1.5 times lower than the 5-year survival rate in the United States^2,3^. The comparatively lower survival and higher mortality rates in China than in countries with higher human development indexes suggest that an efficient and cost-effective cancer prevention and surveillance system is urgently needed. Early detection of cancer is one of the most critical elements in a healthcare system. Early detection of cancer can improve the prognosis and survival at an early stage in nearly all cancer types^4^. Successful screening strategies have led to a significant decline in the incidence and mortality rates of cervical, breast, colorectal, and prostate cancers.

To achieve an early detection of cancer, however, is not an easy task. Investigating the biology and prognosis of early cancer, identifying and validating reliable early detection biomarkers, and developing accessible and accurate early detection technologies have always been the greatest obstacles in the process^4^. Precise detection of cancer can distinguish benign from malignant lesions, which helps avoid unnecessary procedures and facilitates further disease management. Current early detection strategies include endoscope-based biopsies, medical imaging, cytology, immunoassays, and biomarker tests^5–7^. Being intrusive and costly, endoscope-based biopsies carry an inherently heavy burden as a major medical procedure relying on professional personnel. Like cytology, both screening methods depend on medical professionals and are based on personal judgment with a performance that is far from ideal^8^. In contrast, immunoassays are highly inaccurate, given the high false-positive rates. Medical imaging, as a screening tactic, requires expensive equipment and specialized technicians. Hence, medical imaging is extremely limited due to the low accessibility. For all these reasons, biomarkers appear to be a better option for the early detection of cancer.

Biomarkers are currently categorized as proteins, DNA mutation markers, epigenetic markers, chromosomal abnormalities, RNA markers derived directly from tumors, or tumor fragments obtained indirectly from bodily fluids. Protein markers are the most widely applied biomarkers in cancer screening and diagnosis. Protein biomarkers, as screening biomarkers, are limited by the tendency to be affected by benign lesions, which leads to overdiagnosis and overtreatment, as has been reported for α-fetoprotein and prostate-specific antigen (PSA)^9,10^. RNA markers include genetic expression patterns and other non-coding RNA markers. A combination of genetic expression RNA markers can be detected using urine samples, the sensitivity of which was far from satisfactory (60%) for primary tumors, and the detection of which can be affected by the easy degradation nature of RNA in the normal environment^11^. Genetic and epigenetic markers both face the problem of prevalence in tumors and limitation to cancer types.

DNA methylation has been a strong candidate as an early detection biomarker since being first linked to cancer by Feinberg in 1983^12^. DNA methylation aberrations are observed in all stages of cancer, as early as the precancerous stage. Aberrant DNA hypermethylation usually takes place on CpG islands in gene promoters to counteract tumor suppressors^13,14^. Studies have also suggested that abnormal DNA hypermethylation engages in the upregulation of developmental regulators^15^. The DNA methylation valley, which is commonly associated with developmental regulators and hypermethylated cancers, might switch the gene expression mode to a more stable DNA methylation-dependent mode and decrease the connection to methylated histone H3K27me3 and associated polycomb proteins^16,17^.

Among the large number of published DNA methylation markers, several have successfully debuted in the market; however, the current commercialized DNA methylation markers and diagnostic panels have yet to fully unlock the potential of early detection of cancer for multiple reasons^18^. While mostly showing acceptable performance using database information, these biomarkers usually perform less ideally in the real world due to the fact that real-world samples are often more complex and not as representative as those selected in the databases. Next-generation-sequencing-based multi-cancer methylation early detection has been shown to have a mere 16.8% and 40.4% sensitivity in stage I and II cancers, respectively^19^. Early detection tests require greater stability and more accurate biomarkers.

## Universal cancer only marker (UCOM) discovery using guide positioning sequencing (GPS)

Despite decades of cancer research, satisfactory prevention and treatment have not been realized. New methodologies are needed to enable researchers to thoroughly evaluate cancer. Over the last 23 years, 6 cancer hallmarks, such as evading apoptosis, tissue invasion & metastasis, etc., have been expanded to 14 by including features like nonmutational epigenetic reprogramming and polymorphic microbiomes^20,21^. As more details involving cancer are unveiled, more perspectives are introduced into cancer research. Cancer research has gradually come into a new era in two directions (commonality and individuality). With the development of precision oncology in recent years, the focus of cancer research is leaning towards individualized targeted therapy and the heterogeneity of cancer^22^. Thus, recently identified cancer biomarkers have focused mainly on specific cancer types, such as *PAX6* for cervical cancer^23^ and *BMP3* for colorectal cancer^24^. The performance of these biomarkers specific to cancer types varies, but it is still not possible for susceptible individuals to undergo screening for all cancers simultaneously due to the limitation of biological sample acquisition and the high cost. It would be ideal if we could identify a single, robust biomarker that is effective for all types of cancer at an early stage.

To achieve such an ideal goal, a better biomarker candidate must be selected from the list of potential biomarker types. DNA methylation aberrations, among all genetic and epigenetic profiles, are known to be related to cancer and are some of the earliest, if not first, cancer-related abnormalities to occur chronologically. The investigation of DNA methylation started early, but has been hindered by the lack of research methods. Among 28 million potential methylated CpG sites in the genome, a manageable number must be detected and aligned to the genome to better understand tumorigenesis. Whole genome bisulfite sequencing (WGBS), which is considered to be the gold standard of DNA methylation sequencing, can only cover 50% of Cs in cancer cells due to the nature of bisulfite treatment that breaks DNA fragments and lowers the genome complexity during the transformation of Cs-to-Ts^25^. Other methods, such as 450k chips, only cover 1.6% of genome methylation. Based on 450k data, a DNA methylation detection panel has 35.4% sensitivity for 6 types of stage I cancers^26^. Limitations of cancer types, poor performance, and noise generated by detection methods in the analytic process have become the greatest obstacles for pan-cancer detection panels.

**Figure 1:**
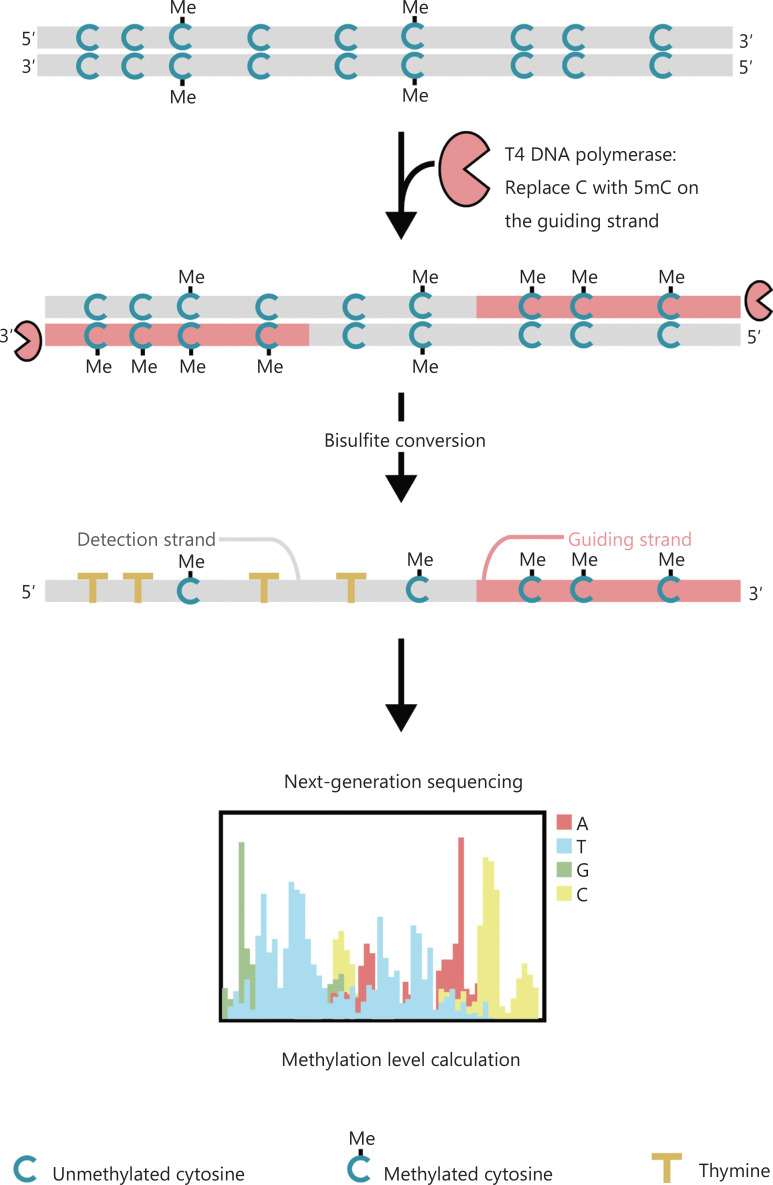
Schematic of GPS workflow for DNA methylation detection^25^. Gray line: input DNA sequence; red line: DNA treated with T4 DNA polymerase, replacing cytosine with 5′-methylcytosine at the 3′ end of the input; blue C with Me: methylated cytosine; blue C: unmethylated cytosine; yellow T: thymine^25^.

To better investigate the epigenetic patterns of cells during tumorigenesis and metastasis, we developed a unique GPS for genome-wide DNA methylation detection, which covers up to 96% of CpG sites in 0.4 billion reads^25^. GPS is a bilateral sequencing method using a 3′ end of DNA fragment of non-convertible methyl-cytosines after bisulfite treatment that guides the alignment of DNA methylation calculation of the 5′ end through pair-end sequencing (**Figure 1**)^25^. The methyl-cytosine guiding strand, acting as a template strand, aids in high-GC region alignment that recovers the most abandoned sequencing data in traditional WGBS. The high coverage feature of GPS provides an enormous amount of DNA methylation information, which allows us to examine cancer methylation profiles with a considerably higher resolution in previously under-investigated regions.

GPS provides us with a powerful tool to investigate the homogeneity of cancer, which can greatly simplify cancer research and potentially find a universal explanation for tumorigenesis and metastasis. While analyzing GPS data of cancer cell lines, a unique phenomenon was frequently encountered. There were a number of regions that appeared to be abnormally hypermethylated in multiple types of cancer samples. This unexpected finding was subsequently validated to serve as UCOMs. Greater than 7,000 samples from 17 types of cancer in The Cancer Genome Atlas (TCGA) database have been analyzed, among which we identified the first UCOM, *HIST1H4F*, a histone-related gene that is hypermethylated in all types of cancer^27^. A series of UCOMs were then found and validated in the TCGA database, the Gene Expression Omnibus (GEO) database, and real-world clinical samples. As of now, *HIST1H4F*, *PCDHGB7*, and *SIX6* have been found and validated as UCOMs. The unexpected discovery of UCOMs offers a powerful answer to the need for early detection of cancer. UCOMs provide a solution for single marker detection of multiple cancers.

## Characteristics of UCOMs

**Figure 2:**
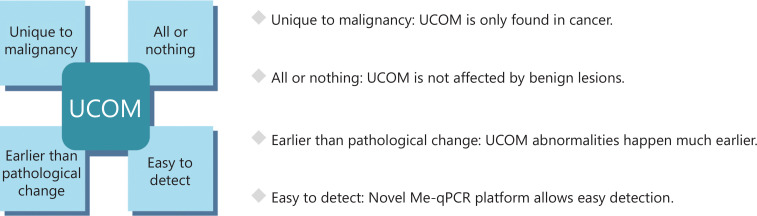
Characteristics of UCOMs.

Upon validation, UCOMs have been shown to exhibit four major characteristics that enable UCOMs to surpass the efficacy of current biomarkers (**Figure 2**).

### Unique to malignancy

UCOMs are unique to cancerous or pre-cancerous lesions and are not affected by normal physiologic changes. Some of the current cancer-related markers that have been widely applied in early detection and/or screening have led to overdiagnosis. Elevated PSA levels, a clinically accredited screening tool, are also detected in benign conditions, such as prostate hyperplasia and prostatitis^10^. The overdiagnosis and resulting overtreatment lead to a reduced quality of life due to bowel, urinary, and sexual complications^28^. Other protein-based and widely used biomarkers in the clinical setting, such as CA-125, have yielded no significant benefits while incurring overdiagnosis and overtreatment^29^. The high specificity of UCOMs for malignancies avoids these shortcomings. The UCOM, *PCDHGB7*, efficiently distinguishes high-grade squamous intraepithelial lesions (HSILs) and cervical cancer from normal samples and low-grade squamous intraepithelial lesions (LSILs), while most other biomarkers can only differentiate cervical cancer from normal samples^30^. Although *PCDHGB7* does not detect significant differences between normal endometrium and endometrial hyperplasia, significant differences are detected between normal endometrium and atypical hyperplasia, and even greater differences are detected between normal endometrium and endometrial cancer (EC) based on *PCDHGB7*^31^. UCOMs are unique to malignant lesions in databases and clinical samples. From a patient’s perspective, unique UCOMs reduce the threshold for understanding complex indications of various poor-performing unstable biomarkers and the corresponding anxiety during the evaluation process. From the clinician’s perspective, unique UCOMs differentiate malignancies from benign lesions, which aid in the triage of patients and reduces unnecessary medical procedures and overtreatment. Therefore, unique UCOMs reduce medical system redundancy, relieve system distress, and make available more medical resources to those in need.

### All or nothing

UCOMs are only present in cancer cells and are detected stably in nearly all cancer cells. *HIST1H4F* was validated to be hypermethylated in nearly all tumor types but not in normal samples^27^. Similarly, *PCDHGB7* and *SIX6* have also been shown to be hypermethylated in all tumor samples but not in normal samples^30–32^. This unique characteristic significantly improves the performance of UCOMs with respect to the limit of detection and sensitivity. As few as 2% of cancer cells can be differentiated in samples, making UCOMs a much more sensitive biomarker than most existing biomarkers^30^. As a biomarker used for colorectal cancer detection, KRAS mutations only exist in approximately 36% of colorectal cancer cases, suggesting poor diagnostic potential^33^. The low prevalence of KRAS mutations in colorectal cancer limits KRAS in combination with other biomarkers. In fact, a combination of biomarkers might seem promising initially, but does not always generate a satisfactory result while demonstrating much greater noise in detection analysis and usually involves more complicated experimental procedures. In contrast, *PCDHGB7* and other UCOMs exist in all cancers. UCOMs detect cancerous components in different types of cancer samples with utmost precision while eradicating complex noise-canceling analysis processes. It is not difficult to detect cancer in an abundant sample, but it is extremely challenging to detect cancer in a small sample. UCOMs are capable of detecting small amounts of cancer.

### Cancer detection preceding pathological changes

UCOMs can be detected in the pre-cancerous stage prior to pathological changes. As epigenetic biomarkers, UCOM abnormalities occur in an earlier stage than phenotypic abnormalities and are detectable throughout tumorigenesis, progression, and metastasis^34,35^. The sensitivity of UCOM over time enhances UCOM performance in detecting early-stage cancer and pre-cancerous lesions. Detection of early cancer based on biopsies and cytology can be difficult for even the most experienced pathologists. A single biopsy acquired *via* colposcopy has been reported as positive in 60.6% of HSIL+ samples. Additional biopsies are required for multiple lesions to increase sensitivity^36^. In contrast, the UCOM, *PCDHGB7*, has a sensitivity of 82% for HSIL+ samples, surpassing the sensitivity of biopsies and most biomarkers^30^. The methylation marker, *FAM19A4*, has a sensitivity of 69% for CIN2+, which is similar to cytology, but cannot differentiate CIN1 from normal samples^37^. UCOMs have been shown to be a much more sensitive early detection biomarker. Compared with experience-based pathologists, UCOMs have superior detection sensitivity for early-stage cancers, which in turn contributes to improved cancer prognosis and survival^30^. Additionally, UCOMs offer a detection platform that is accessible to areas lacking experienced pathologists and greatly improves detection efficiency. With uniform sampling and detection procedures, UCOM detection yields stable and easy-to-interpret results that better suit a screening protocol requiring fewer professional personnel and medical resources.

### Easy to detect

**Figure 3:**
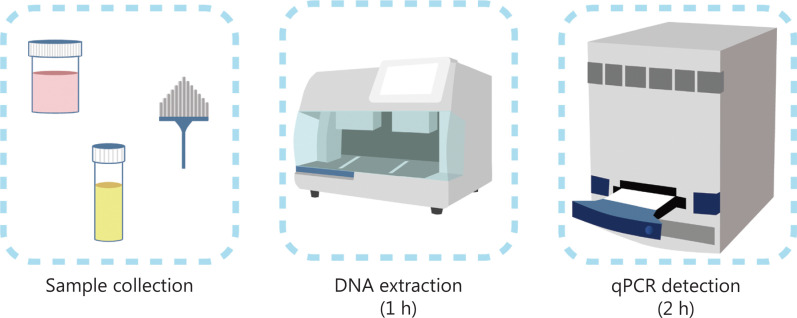
Detection process of UCOMs. Sample types include professionally sampled BALF, Pap brush, and/or self-collected urine. The DNA extraction process can be accommodated to an automatic extractor, the product of which can be directly detected by qPCR.

Current methods for DNA methylation detection are complicated and time-consuming. Most of the methods require bisulfite transformation, which causes a loss in sample quality and possibly produces unstable and inaccurate results. The poor reproducibility caused by bisulfite treatment potentially leads to confusion for physicians and patients and further interfere with the follow-up and/or treatment strategies. Therefore, we further modified the method of UCOM detection to avoid problematic bisulfite treatment of the samples, accommodate the clinical application requirements, and enhance accessibility. We developed a novel method using methylation-sensitive restriction enzymes combined with real-time fluorescent quantitative PCR (Me-qPCR) to quantify the methylation status of UCOMs within 3 h using easy handling procedures (**Figure 3**). Me-qPCR can accommodate multiple sample types, such as clinical collection of body fluids and self-collected urine samples. Collected clinical samples can be processed, stored, and easily proceed to detection using standardized and automated DNA extraction. The extracted DNA can then be directly applied to the Me-qPCR platform for a one-pot reaction and output quantification results. After simple result analysis using diagnostic models fitted and validated to specific cancer types, the final determination of UCOM detection results is interpreted and presented as a semi-quantitative value. The Me-qPCR platform outperforms the traditional bisulfite-pyrosequencing in UCOM detection while saving 3 h of bisulfite conversion, according to the EZ DNA Methylation-Gold kit protocol. The innovative methylation detection platform makes UCOM detection stabler, more accurate, and more accessible^30^.

## Application of UCOMs

### Lung cancer

Lung cancer is the second most frequently diagnosed and most lethal cancer worldwide, accounting for 11.4% of new cases and 18.0% of new deaths^1^. Among all diagnoses, 85% are non-small cell lung cancer (NSCLC) and 15% are small cell lung cancer (SCLC), which has a higher level of malignancy^38^. Low-dose computed tomography (LDCT) scanning is the currently recommended screening method for lung cancer and has been shown to improve early detection and reduce mortality^6^; however, due to low specificity and poor accessibility, LDCT has yet to serve as a satisfactory screening method, as do other common cancer markers, such as CEA^39^. The costs and potential for missed diagnoses and misdiagnoses of the LDCT screening strategy impede the progress of lung cancer screening promotion^40^.

**Figure 4:**
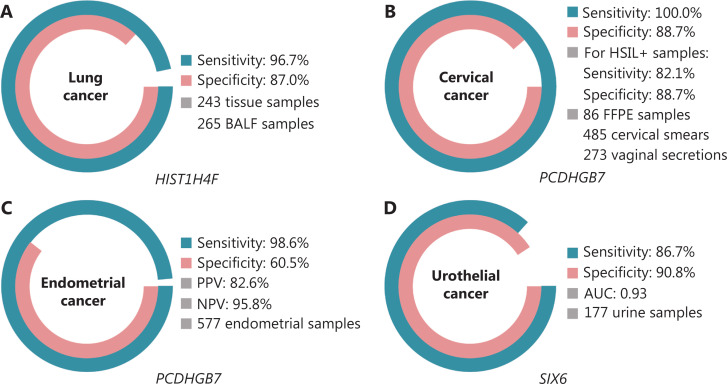
UCOMs have been validated in four types of cancer in large-scale clinical studies. A. Performance of *HIST1H4F*, a UCOM, in lung cancer detection of 508 samples. B. Performance of *PCDHGB7*, a UCOM, in cervical cancer detection of 844 samples. C. Performance of *PCDHGB7*, a UCOM, in endometrial cancer detection of 577 endometrial Pap and Tao brush samples. D. Performance of *SIX6*, a UCOM, in urothelial cancer detection of 177 samples.

*HIST1H4F*, a UCOM, has enormous potential as an early detection biomarker in bronchoalveolar fluid (BALF) samples^27^. *HIST1H4F* is hypermethylated in lung adenocarcinoma and lung squamous cell carcinoma, with a detection specificity of 96.7% and sensitivity of 87.0% (**Figure 4A**), and an exceptional performance for stage I cancers^27^. *HIST1H4F* has a specificity of 96.5% and a sensitivity of 85.4% for NSCLC, and 96.5% and 95.7%, respectively, for SCLC^27^. Additionally, samples of eight other types of cancer, including pancreatic and colorectal cancers, have validated that *HIST1H4F* is hypermethylated in all eight types^27^.

### Cervical cancer

Cervical cancer was the fourth most frequently diagnosed cancer and the fourth leading cause of cancer deaths in women in 2020, accounting for 3.1% of new cases and 3.4% of cancer-related deaths globally^1^. To eliminate cervical cancer by 2030, as proposed by the WHO, early detection of cervical cancer is a necessity. If detected at an early stage, the 5-year survival rate reaches 92% with invasive cervical cancer^41^. The American Cancer Society (ACS) guidelines suggest cervical cytology tests, primary HPV tests, or cotests for screening^42^. Cervical cytology is invasive and can only detect 63.5% of CIN2+ cases^37^.

*PCDHGB7*, in contrast, has performed much better using Pap smears and vaginal secretions, and can efficiently differentiate HSIL from LSIL in an ultra-early stage. *PCDHGB7* alone has a sensitivity of 100.0% and a specificity of 88.7% for cervical cancer (**Figure 4B**), and an 82.1% sensitivity and 88.7% specificity for HSIL+ samples^30^. *PCDHGB7* also has a 90.9% sensitivity and 90.4% specificity in vaginal secretion samples for cervical cancer, which are much easier to collect^30^. When combined with the high-risk (hr)HPV test or Thinprep Cytology Test (TCT), *PCDHGB7* has an increased sensitivity of 95.7% and specificity of 96.2%, significantly surpassing that of the hrHPV test (20.3%), TCT (51.2%), and the two combined (57.8%) for cervical cancer^30^. *PCDHGB7* has also been shown to be hypermethylated in 17 types of cancer from the TCGA database, indicating its suitability in the UCOM family^30^.

### EC

EC is one of the most common female reproductive system cancers worldwide, with an estimated 4.2 million new cases and 1% of cancer-related deaths annually^1^. With a successful diagnosis at an early stage, EC is curable and has a 5-year survival rate of 95% for stage I cancer. Patients who are symptomatic, such as abnormal uterine bleeding, receive periodic clinical evaluation and undergo invasive and painful biopsy procedures, despite only 5%–10% eventually developing EC^43^. Transvaginal ultrasound, as the common detection method, is highly unreliable due to its inability to differentiate benign from malignant endometrial changes and the high false-positive rate^44^.

A parallel comparison of serum CA-125, a widely implemented EC biomarker, and *PCDHGB7* was conducted. Serum CA-125 had a sensitivity of 24.8%, which suggests that CA-125 is an inadequate marker for EC despite a specificity of 92.3%^31^. *PCDHGB7* detection using Pap brush samples yielded a sensitivity of 80.65% and a specificity of 82.81% for EC at all stages, while a Tao brush had a sensitivity of 61.29% and a specificity of 95.31%^31^. The *PCDHGB7* diagnostic model, based on Me-qPCR, yielded a sensitivity of 98.61%, a specificity of 60.5%, and an overall accuracy of 85.5%, using Pap and Tao brush samples (**Figure 4C**)^31^.

### Urothelial cancer

Urothelial cancer, consisting of bladder, renal pelvis, and ureter cancers, was the seventh most frequently diagnosed cancer in 2020 worldwide, causing 5.2% of new cases and 3.9% of deaths^1^. Urothelial cancers, greater than 50% of which are bladder cancer, were the fourth most frequently diagnosed cancer in the United States in 2022, accounting for 11.6% of newly diagnosed cases^3^. Approximately 75% of bladder cancers are classified as non-muscle invasive bladder cancer restricted to the mucosa or submucosa^45^. A cystoscopy biopsy is the gold standard for diagnosing urothelial cancer implemented by fluorescence *in situ* hybridization (FISH) and cytology tests. FISH and cytology have poor diagnostic performance, and cystoscopy is intrusive and has the underlying risk of missing microlesions, misinterpreting lesions, and potentially causing a spread or relapse of cancer^46^.

The previously validated UCOM, *PCDHGB7*, was also shown to be hypermethylated in urothelial cancer, with an area under the curve of 0.86, suggesting a potential diagnostic capability^30^. To further validate more UCOMs and better accommodate more sample types, *SIX6*, a novel UCOM, was examined and showed excellent diagnostic potential in the early detection of urothelial cancer using urine samples on the Me-qPCR platform. *SIX6* detection using urine samples demonstrated a competitive sensitivity of 86.7% and a specificity of 90.8% (**Figure 4D**), while being non-invasive and easy to acquire^32^. The potential of *SIX6* in metastasis monitoring and treatment efficacy evaluation is currently under investigation.

## The future and challenges

UCOMs have a strong performance in the diagnostic potential of multiple cancers, but there is much work left to do. We have been expanding the list of UCOMs and have been actively validating UCOMs in more types of cancer, including those that are traditionally difficult to detect. Validation results from TCGA databases have further corroborated the application of UCOMs in more types of cancer and more situations. In a preliminary investigation, UCOMs have been shown to have robust diagnostic potential for cholangiocarcinomas and pancreatic adenocarcinomas, which are nearly impossible to diagnose in an early stage with current screening methods^32,47^. The ability to detect rare cancers with UCOMs can be utilized with circulating tumor DNA (ctDNA) by an improved liquid biopsy platform^48^. A study involving a plasma DNA-based pan-cancer detection panel yielded a sensitivity of 57.9%^49^. Despite the high specificity, the overall performance reveals that there is still room for improvement.

The unique characteristics of UCOMs have also supported the investigation of UCOM potential in treatment efficacy evaluation and recurrence monitoring. According to the Response Evaluation Criteria in Solid Tumours (RECIST), medical imaging is the recommended methodology for recurrence monitoring and treatment efficacy evaluation, while tumor markers are used alone for assessment^50^. In reality, however, imaging approaches are greatly affected by the frequency and timing, and therefore expose patients to higher risk and costs^51,52^. *SIX6* has been validated to serve as a predictor for breast cancer metastasis^32^. Liquid biopsy-based ctDNA monitoring enables real-time surveillance over minimal residual disease months ahead of radiologic detection, ideally delaying and preventing relapse-related cancer progression^53^. Preliminary results suggest that UCOMs reflect the level of cancerous hypermethylation in real time immediately after surgery and treatment^32^. The high sensitivity exhibited by UCOMs and the applicability in multiple non-intrusive sample types allows UCOMs to serve as a precise recurrence monitoring biomarker while maintaining high patient compliance.

At the same time, public accessibility to the test is another major issue that requires additional effort. While UCOM detection collaborations have been adopted in more hospitals in the hope of benefiting more patients, *pro bono* detections and screenings have been actively performed in rural China. UCOMs require improved accessibility to qualify as a feasible screening tool, especially for underdeveloped areas.

While the UCOM application results in early detection are promising, many unknowns about UCOM exist. With active exploration, additional research is warranted about why UCOMs are universally present in cancers. The underlying epigenetic regulation mechanisms underlying UCOMs are worthy of further investigation, which could justify a new direction for cancer therapeutics. Returning to the interplay between tumor homogeneity and heterogeneity, we are interested in why UCOMs can be an exception to the majority of cancer biomarkers that are tightly linked to specific cancer types. The role of UCOM-identified DNA methylation aberrations in tumorigenesis, tumor progression, and metastasis has not been determined in the process of losing and regaining cell identity and necessitates a thorough inspection. Another major interest lies in the scope of the incorporation of the homogeneity trait of UCOMs with tissue-unique markers in the hope of approaching precise detection of cancer traces and identification of tumor tissue origins in a reverse manner. UCOMs can be an ideal tool to prevent cancer, detect cancer, and potentially defend and eliminate cancer.

## References

[1] Sung H, Ferlay J, Siegel RL, Laversanne M, Soerjomataram I, Jemal A (2021). Global Cancer Statistics 2020: GLOBOCAN estimates of incidence and mortality worldwide for 36 cancers in 185 countries. CA Cancer J Clin.

[2] Xia C, Dong X, Li H, Cao M, Sun D, He S (2022). Cancer statistics in China and United States, 2022: profiles, trends, and determinants. Chin Med J (Engl).

[3] Siegel RL, Miller KD, Wagle NS, Jemal A (2023). Cancer statistics, 2023. CA Cancer J Clin.

[4] Crosby D, Bhatia S, Brindle KM, Coussens LM, Dive C, Emberton M (2022). Early detection of cancer. Science.

[5] Ladabaum U, Dominitz JA, Kahi C, Schoen RE (2020). Strategies for colorectal cancer screening. Gastroenterology.

[6] Tanoue LT, Tanner NT, Gould MK, Silvestri GA (2015). Lung cancer screening. Am J Respir Crit Care Med.

[7] Bouvard V, Wentzensen N, Mackie A, Berkhof J, Brotherton J, Giorgi-Rossi P (2021). The IARC perspective on cervical cancer screening. N Engl J Med.

[8] Xue P, Ng MTA, Qiao Y (2020). The challenges of colposcopy for cervical cancer screening in LMICs and solutions by artificial intelligence. BMC Med.

[9] Johnson P, Zhou Q, Dao DY, Lo YMD (2022). Circulating biomarkers in the diagnosis and management of hepatocellular carcinoma. Nat Rev Gastroenterol Hepatol.

[10] Van Poppel H, Albreht T, Basu P, Hogenhout R, Collen S, Roobol M (2022). Serum PSA-based early detection of prostate cancer in Europe and globally: past, present and future. Nat Rev Urol.

[11] Holyoake A, O’Sullivan P, Pollock R, Best T, Watanabe J, Kajita Y (2008). Development of a multiplex RNA urine test for the detection and stratification of transitional cell carcinoma of the bladder. Clin Cancer Res.

[12] Feinberg AP, Vogelstein B (1983). Hypomethylation distinguishes genes of some human cancers from their normal counterparts. Nature.

[13] Ng JM, Yu J (2015). Promoter hypermethylation of tumour suppressor genes as potential biomarkers in colorectal cancer. Int J Mol Sci.

[14] Esteller M (2007). Cancer epigenomics: DNA methylomes and histone-modification maps. Nat Rev Genet.

[15] Nishiyama A, Nakanishi M (2021). Navigating the DNA methylation landscape of cancer. Trends Genet.

[16] Xie W, Schultz MD, Lister R, Hou Z, Rajagopal N, Ray P (2013). Epigenomic analysis of multilineage differentiation of human embryonic stem cells. Cell.

[17] Li Y, Zheng H, Wang Q, Zhou C, Wei L, Liu X (2018). Genome-wide analyses reveal a role of Polycomb in promoting hypomethylation of DNA methylation valleys. Genome Biol.

[18] Koch A, Joosten SC, Feng Z, de Ruijter TC, Draht MX, Melotte V (2018). Analysis of DNA methylation in cancer: location revisited. Nat Rev Clin Oncol.

[19] Klein EA, Richards D, Cohn A, Tummala M, Lapham R, Cosgrove D (2021). Clinical validation of a targeted methylation-based multi-cancer early detection test using an independent validation set. Ann Oncol.

[20] Hanahan D, Weinberg RA (2000). The hallmarks of cancer. Cell.

[21] Hanahan D (2022). Hallmarks of cancer: new dimensions. Cancer Discov.

[22] Schwartzberg L, Kim ES, Liu D, Schrag D (2017). Precision oncology: who, how, what, when, and when not?. Am Soc Clin Oncol Educ Book.

[23] Liu H, Meng X, Wang J (2020). Real time quantitative methylation detection of PAX1 gene in cervical cancer screening. Int J Gynecol Cancer.

[24] Imperiale TF, Ransohoff DF, Itzkowitz SH, Levin TR, Lavin P, Lidgard GP (2014). Multitarget stool DNA testing for colorectal-cancer screening. N Engl J Med.

[25] Li J, Li Y, Li W, Luo H, Xi Y, Dong S (2019). Guide positioning sequencing identifies aberrant DNA methylation patterns that alter cell identity and tumor-immune surveillance networks. Genome Res.

[26] Gao Q, Lin YP, Li BS, Wang GQ, Dong LQ, Shen BY (2023). Unintrusive multi-cancer detection by circulating cell-free DNA methylation sequencing (THUNDER): development and independent validation studies. Ann Oncol.

[27] Dong S, Li W, Wang L, Hu J, Song Y, Zhang B (2019). Histone-related genes are hypermethylated in lung cancer and hypermethylated HIST1H4F could serve as a pan-cancer biomarker. Cancer Res.

[28] Heijnsdijk EA, Wever EM, Auvinen A, Hugosson J, Ciatto S, Nelen V (2012). Quality-of-life effects of prostate-specific antigen screening. N Engl J Med.

[29] Luzak A, Schnell-Inderst P, Bühn S, Mayer-Zitarosa A, Siebert U (2016). Clinical effectiveness of cancer screening biomarker tests offered as self-pay health service: a systematic review. Eur J Public Health.

[30] Dong S, Lu Q, Xu P, Chen L, Duan X, Mao Z (2021). Hypermethylated PCDHGB7 as a universal cancer only marker and its application in early cervical cancer screening. Clin Transl Med.

[31] Yuan J, Mao Z, Lu Q, Xu P, Wang C, Xu X (2022). Hypermethylated PCDHGB7 as a biomarker for early detection of endometrial cancer in endometrial brush samples and cervical scrapings. Front Mol Biosci.

[32] Dong S, Yang Z, Xu P, Zheng W, Zhang B, Fu F (2022). Mutually exclusive epigenetic modification on SIX6 with hypermethylation for precancerous stage and metastasis emergence tracing. Signal Transduct Target Ther.

[33] Huang L, Guo Z, Wang F, Fu L (2021). KRAS mutation: from undruggable to druggable in cancer. Signal Transduct Target Ther.

[34] Belinsky SA, Nikula KJ, Palmisano WA, Michels R, Saccomanno G, Gabrielson E (1998). Aberrant methylation of p16(INK4a) is an early event in lung cancer and a potential biomarker for early diagnosis. Proc Natl Acad Sci U S A.

[35] Robertson KD (2005). DNA methylation and human disease. Nat Rev Genet.

[36] Wentzensen N, Walker JL, Gold MA, Smith KM, Zuna RE, Mathews C (2015). Multiple biopsies and detection of cervical cancer precursors at colposcopy. J Clin Oncol.

[37] De Strooper LM, Meijer CJ, Berkhof J, Hesselink AT, Snijders PJ, Steenbergen RD (2014). Methylation analysis of the FAM19A4 gene in cervical scrapes is highly efficient in detecting cervical carcinomas and advanced CIN2/3 lesions. Cancer Prev Res (Phila).

[38] Thai AA, Solomon BJ, Sequist LV, Gainor JF, Heist RS (2021). Lung cancer. Lancet.

[39] Grunnet M, Sorensen JB (2012). Carcinoembryonic antigen (CEA) as tumor marker in lung cancer. Lung Cancer.

[40] Wood DE, Kazerooni EA, Baum SL, Eapen GA, Ettinger DS, Hou L (2018). Lung Cancer Screening, Version 3.2018, NCCN Clinical Practice Guidelines in Oncology. J Natl Compr Canc Netw.

[41] American Cancer Society (2023). Cancer facts & figures.

[42] Fontham ETH, Wolf AMD, Church TR, Etzioni R, Flowers CR, Herzig A (2020). Cervical cancer screening for individuals at average risk: 2020 guideline update from the American Cancer Society. CA Cancer J Clin.

[43] Clarke MA, Long BJ, Del Mar Morillo A, Arbyn M, Bakkum-Gamez JN, Wentzensen N (2018). Association of endometrial cancer risk with postmenopausal bleeding in women: a systematic review and meta-analysis. JAMA Intern Med.

[44] Jacobs I, Gentry-Maharaj A, Burnell M, Manchanda R, Singh N, Sharma A (2011). Sensitivity of transvaginal ultrasound screening for endometrial cancer in postmenopausal women: a case-control study within the UKCTOCS cohort. Lancet Oncol.

[45] Babjuk M, Burger M, Compérat EM, Gontero P, Mostafid AH, Palou J (2019). European Association of Urology Guidelines on Non-muscle-invasive Bladder Cancer (TaT1 and Carcinoma In Situ) - 2019 Update. Eur Urol.

[46] Aragon-Ching JB (2017). Challenges and advances in the diagnosis, biology, and treatment of urothelial upper tract and bladder carcinomas. Urol Oncol.

[47] Rizvi S, Khan SA, Hallemeier CL, Kelley RK, Gores GJ (2018). Cholangiocarcinoma – evolving concepts and therapeutic strategies. Nat Rev Clin Oncol.

[48] Ye Q, Ling S, Zheng S, Xu X (2019). Liquid biopsy in hepatocellular carcinoma: circulating tumor cells and circulating tumor DNA. Mol Cancer.

[49] Zhang Y, Yao Y, Xu Y, Li L, Gong Y, Zhang K (2021). Pan-cancer circulating tumor DNA detection in over 10,000 Chinese patients. Nat Commun.

[50] Eisenhauer EA, Therasse P, Bogaerts J, Schwartz LH, Sargent D, Ford R (2009). New response evaluation criteria in solid tumours: revised RECIST guideline (version 1.1). Eur J Cancer.

[51] Litière S, Collette S, de Vries EG, Seymour L, Bogaerts J (2017). RECIST - learning from the past to build the future. Nat Rev Clin Oncol.

[52] Seymour L, Bogaerts J, Perrone A, Ford R, Schwartz LH, Mandrekar S (2017). iRECIST: guidelines for response criteria for use in trials testing immunotherapeutics. Lancet Oncol.

[53] Pantel K, Alix-Panabières C (2019). Liquid biopsy and minimal residual disease – latest advances and implications for cure. Nat Rev Clin Oncol.

